# Focal Bronchiectasis Causing Abnormal Pulmonary Radioiodine Uptake in a Patient with Well-Differentiated Papillary Thyroid Carcinoma

**DOI:** 10.1155/2012/452758

**Published:** 2012-10-11

**Authors:** Ash Gargya, Elizabeth Chua

**Affiliations:** Department of Endocrinology, Royal Prince Alfred Hospital, NSW 2050, Australia

## Abstract

*Background*. False-positive pulmonary radioactive iodine uptake in the followup of differentiated thyroid carcinoma has been reported in patients with certain respiratory conditions. *Patient Findings*. We describe a case of well-differentiated papillary thyroid carcinoma treated by total thyroidectomy and radioiodine ablation therapy. Postablation radioiodine whole body scan and subsequent diagnostic radioiodine whole body scans have shown persistent uptake in the left hemithorax despite an undetectable stimulated serum thyroglobulin in the absence of interfering thyroglobulin antibodies. Contrast-enhanced chest computed tomography has confirmed that the abnormal pulmonary radioiodine uptake correlates with focal bronchiectasis. *Summary*. Bronchiectasis can cause abnormal chest radioactive iodine uptake in the followup of differentiated thyroid carcinoma. *Conclusions*. Recognition of potential false-positive chest radioactive iodine uptake, simulating pulmonary metastases, is needed to avoid unnecessary exposure to further radiation from repeated therapeutic doses of radioactive iodine.

## 1. Introduction

False-positive pulmonary radioactive iodine uptake in the followup of differentiated thyroid carcinoma has been reported in patients with certain respiratory conditions.

## 2. Patient

**Figure 1:**
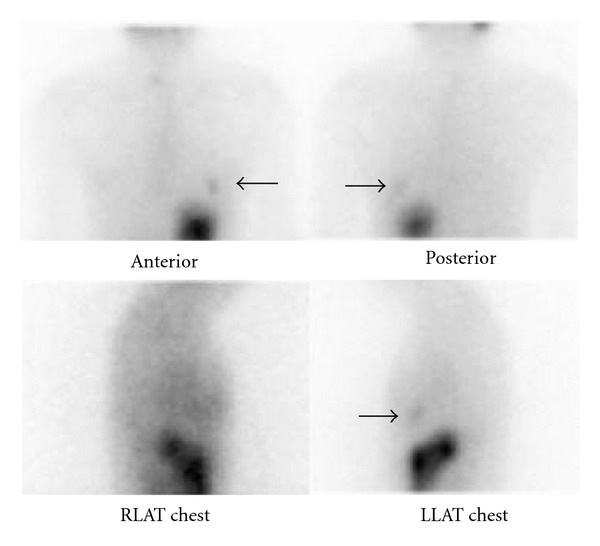
Whole-body scan immediately following 100 mCi (3700 MBq) thyroid remnant ablation showing uptake in thyroid bed and left hemithorax.

**Figure 2:**
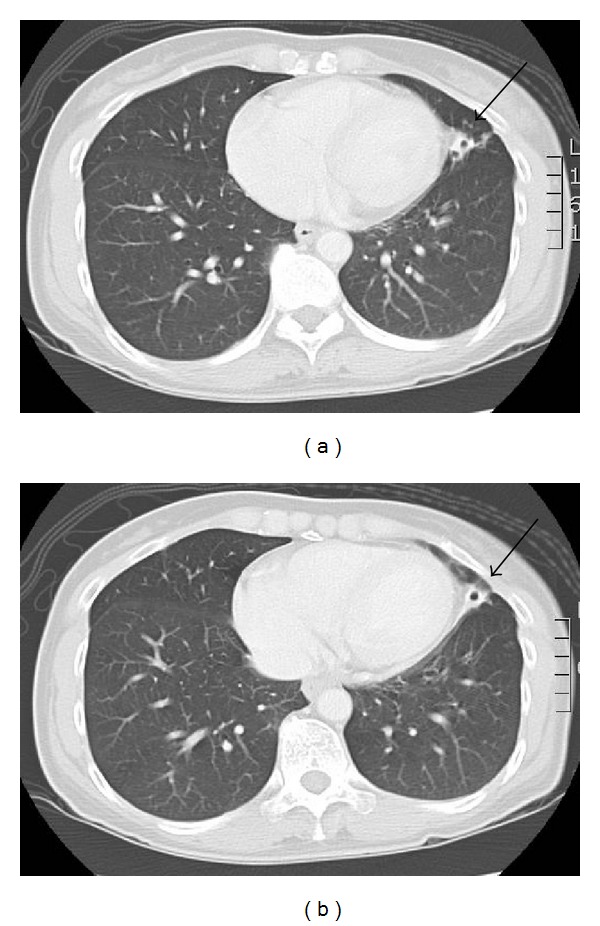
Computerised tomography of the chest performed initially (a) and after 2 years (b) showing focal bronchiectasis in the lingula of the left lung.

A 46-year-old woman was diagnosed two years ago with T2N0M0 papillary thyroid carcinoma that was managed with total thyroidectomy and radioiodine (RAI) ablation therapy (100 mCi or 3700 MBq). The postablation RAI-whole body scan showed uptake in the thyroid bed and left hemithorax (see Figure 1). A contrast-enhanced chest computed tomography (CT) performed six months later showed a left 1.5 × 1.4-cm cavitating lesion in the lingula of the left lung (see Figure 2). This was reviewed by a respiratory physician who confirmed that the lesion was consistent with focal bronchiectasis.

**Figure 3:**
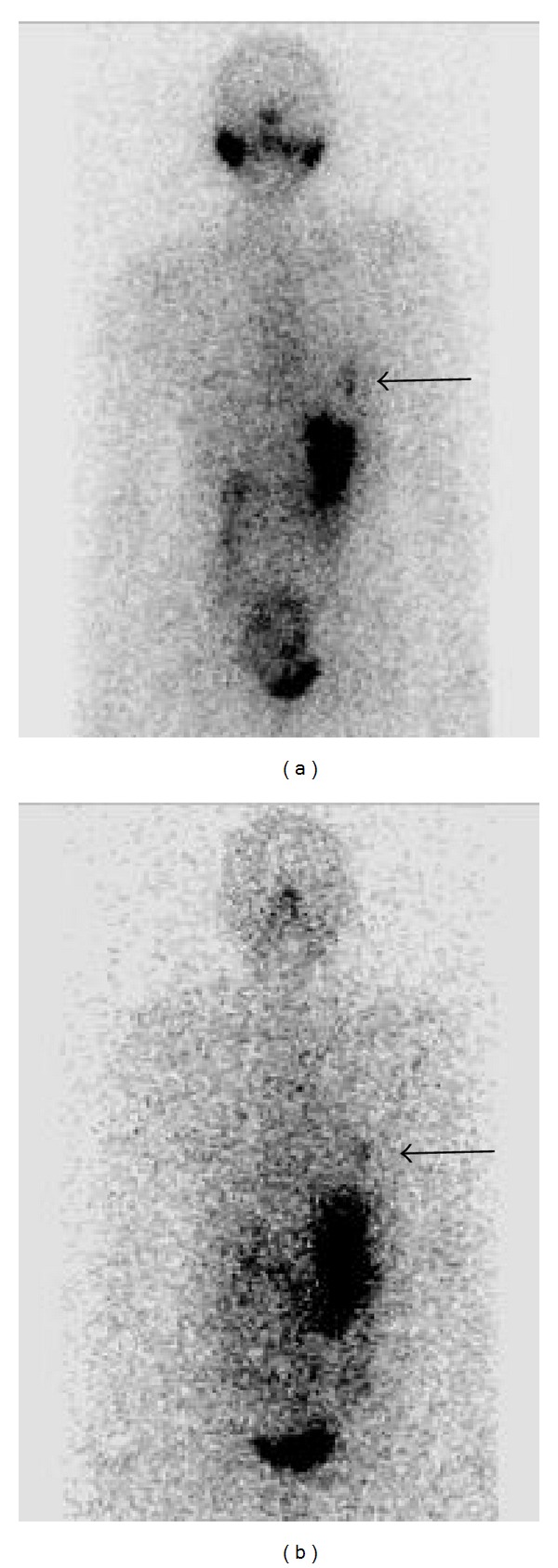
Follow-up diagnostic I-123 TSH-stimulated whole-body scan (anterior views) performed one year (a) and two years (b) after thyroid remnant ablation showing persistent uptake in the left hemithorax.

Subsequent TSH-stimulated diagnostic RAI-whole body scans performed one and two years after the initial RAI-ablation have shown persistent uptake in the left hemithorax alone (see Figure 3) despite an undetectable stimulated serum thyroglobulin of <0.2 ug/L (with no interfering thyroglobulin antibodies). Neck ultrasounds have been negative for metastases.

## 3. Discussion

False-positive chest RAI uptake can be seen in patients with acute respiratory tract infections, chronic pulmonary inflammation, primary lung tumours, fungal infections, rheumatoid-associated lung disease, and inactive pulmonary tuberculosis [1, 2]. Previous case reports of bronchiectasis causing abnormal pulmonary radioiodine uptake have been described [2–6]. Possible explanations for the abnormal RAI uptake seen in patients with chronic pulmonary inflammation include (a) the concentration of iodide salt due to increased vascularity and capillary permeability at inflamed mucosal surfaces and/or (b) the accumulation of tracheobronchial inflammatory exudate in damaged lung regions [1]. Recognition of potential false-positive chest RAI uptake, simulating pulmonary metastases, is needed to avoid unnecessary exposure to further radiation from repeated therapeutic doses of RAI.
